# Does one heavy load back squat set lead to postactivation performance enhancement of three-point explosion and sprint in third division American football players?

**DOI:** 10.1186/s13102-021-00288-y

**Published:** 2021-06-07

**Authors:** Robert Bielitzki, Daniel Hamacher, Astrid Zech

**Affiliations:** 1 Department of Sport Science, Institute III, Otto von Guericke University Magdeburg, Magdeburg, Germany; 2 Institute of Sport Science, Friedrich Schiller University Jena, Jena, Germany

**Keywords:** postactivation potentiation, conditioning contraction, sprint performance, squat, blocking

## Abstract

**Background:**

American football players need the ability to provide maximal muscular power in a modicum of time. Postactivation performance enhancement (PAPE), which is characterized by an acute improvement of a performance measure following conditioning contractions, could be of value for American football players. The aim of the present study was to determine the effect of a heavy load back squat PAPE protocol on three-point explosion (TPE; an essential blocking technique and drill) and 40-yard dash (40YD) performance compared to a traditional warm-up in American football players.

**Methods:**

In a crossover study design, eighteen male competitive regional league American football players (mean ± SD: body mass 93.9 ± 15.5 kg, height 181.4 ± 6.8 cm, age 24.8 ± 3.9 years) performed a TPE on a double blocking sled (weight: 150 kg) and a 40YD (36.6 m with a 5 and 10 m split) 8 min after two different warm-up conditions. One condition was a traditional, football specific warm-up (TWU) consisting of game related movements (e.g. backward lunges, lateral power steps), whereas the other condition (PAPE) consisted of three explosive back squats with a load of 91 % one-repetition maximum.

**Results:**

There was no significant difference in TPE between TWU and PAPE. For the 40YD, we found significantly shorter sprint times in the PAPE condition with medium effect sizes for the 5 m (*p* = 0.007; *r* = 0.45) and 10 m (*p* = 0.020; *r* = 0.39) but not for the whole 36.6 m distance (*p* = 0.084; *r* = 0.29) compared to the TWU condition.

**Conclusions:**

The used heavy load back squat PAPE protocol improved sprint performance over short distances (≤ 10 m) but not complex movements like the three-point explosion.

## Introduction

In various sports, one of the most important abilities is the production of maximal muscular power [1]. Different strategies are used to acutely increase the performance during power-based exercises. These conditioning activities include exercises performed with low and high load/intensity. The latter one has gained increasing attention in the last years and refers to an increased performance following a maximum or near maximum conditioning contraction, which is defined as postactivation performance enhancement (PAPE) [2]. To date, the key mechanisms of PAPE are still not clear as there are various contributing factors (e.g. muscle temperature, muscle and muscle fiber water content, muscle activation) [2, 3].

Past studies confirmed that PAPE protocols are effective in athletes with long-term experience in resistance training (> 3 years [4]) and greater maximum muscle strength [5–7]. However, conflicting results with negative as well as no effects were revealed for less experienced athletes [8]. Therefore, PAPE protocols should be carefully used since individual characteristics of athletes can affect subsequent performance. Beneficial effects of PAPE protocols were shown for horizontal [9, 10] vertical jump [10–12], sprint [13, 14] as well as upper-body ballistic activities [10, 15, 16]. These studies included ski jumpers [17], lugers [18], fencers [19] and team sport athletes, e.g. soccer [20], basketball [11, 18], volleyball [11, 21] and rugby [22–24].

According to the current state of research, it can be suggested that American football players might be very sensitive to PAPE because of their physical characteristics and sport specific requirements [25] containing short explosive power and strength elements including sprinting, tackling and blocking [26]. To date, only insufficient empirical data exist for the effect of PAPE protocols in American football players [10, 27, 28]. For example, Tano and colleagues [28] found significant improvements in 20-yard sprint and sled push following a conditioning contraction compared to a dynamic warm-up in high school football players. Furthermore, Evetovich et al. [10] compared different warm-up strategies in NCAA Division II football players with improvements in 36.6 m sprint (40 yards) but not in vertical jump performance following heavy load back squats. Nevertheless, it is questionable whether these effects can be transferred to sport specific movements. Therefore, the objective of this study was to determine the effects of a heavy load back squat PAPE against a traditional warm-up on two sport specific performance measures in American football players. These included the three-point explosion (TPE) as an essential blocking technique as well as the 40-yard dash (40YD). We hypothesized that the heavy load back squat PAPE protocol improves TPE and sprint performance compared to the traditional warm-up in American football players.

## Methods

### Participants

Eighteen male American football players (Table 1) from a regional league team in Germany (third division) voluntarily agreed in writing to participate in the present study. All data was gathered during the regular season (May – July 2019). The inclusion criteria for this study were experience in muscle strength training and American football training for at least one year, regular participation in American football practice and games in the past year and the ability to perform the squat movement and the TPE with an adequate technique. The following positions were included: offensive lineman, defensive lineman, defensive back, linebacker and wide receiver. Participants with any cardiovascular or respiratory disease and subjective record of musculoskeletal injury were excluded. The eligibility of subjects was assessed by interview. The study received approval by the Ethics Committee of the Faculty of Social and Behavioural Sciences, Friedrich Schiller University Jena conforming to the principles of the Declaration of Helsinki on human experimentation.

**Table 1:** Participants’ physical characteristics (*n* = 18)

Variables	Mean ± SD
Body mass (kg)	93.9 ± 15.5
Height (cm)	181.4 ± 6.8
Age (years)	24.8 ± 3.9
3RM Squat (kg)	122.3 ± 15.2
1RM Squat^a^ (kg)	131.7 ± 16.4
Experience in resistance training (years)	3.8 ± 3.0
Experience in American football (years)	3.8 ± 0.2

^a^one-repetition maximum (1RM) estimated from subjects’ three-repetition maximum (3RM) strength testing (Baechle & Earle, 2008)

### Experimental Procedures

A counterbalanced randomized crossover study design was used to compare the effect of a traditional, American football specific warm-up (TWU) with a PAPE intervention on a blocking technique and sprinting performance with a wash-out period of 1 week. In a first laboratory test session, the back squat three-repetition maximum (3RM) was determined for each athlete. For the following two test sessions, the participants were randomly assigned to the PAPE and TWU intervention. During both conditions, participants started with a standardized specific group warm-up followed by two trials of the TPE and 40YD. Participants of both groups then completed a light intensity run for 5 min around the football field. The subsequent warm-up routine differed between conditions. During the TWU condition, subjects started with a traditional warm-up for 5 min consisting of American football specific movements (e.g. backward lunges, lateral power step, shuffle to sprint) while they performed a heavy load resistance exercise of one set of three back squat repetitions at 91 % one-repetition maximum (1RM) during the PAPE condition [23]. In agreement with current evidence [7, 29], players were instructed to perform shallower squats that seem to have greater potentiation effects than deeper squats. After a resting period of 8 min [13, 22], the participants executed the TPE and 40YD. Both tests were performed in an optimal recovery window between 8 and 12 min after the warm-up procedures [30, 31]. Both groups were repeatedly tested on another day using the warm-up condition. Participants were instructed to avoid strenuous exercise, caffeine and alcohol for 48 h prior to the testing sessions. The consumption of a maximal water intake of 500 ml was allowed during the tests. All testing sessions were conducted on the match field of the team at an air temperature between 20 and 26 °C.

### Measurements

#### Three-repetition maximum squat muscle strength

The 3RM squat testing procedure was performed according to Kilduff et al. [22] and Bevan et al. [23]. At first, anthropometric data were ascertained involving measurements of height and body mass. Before strength testing, all athletes completed a standardized warm-up consisting of 5 min light intensity cycling and dynamic exercises including the muscle groups that were needed for the squat movement. Subsequently, three warm-up sets with 8 repetitions at 50 % 1RM, 4 repetitions at 70 % 1RM and finally 2 repetitions at 80 % 1RM were performed. The initial 1RM was self-reported by using data from training diaries. For strength testing, each athlete was instructed to perform 3 repetitions of a set load (3RM). If the trial was successful, the weight was increased until the individual was incapable to lift the weight through the full range of motion. A rest interval of 5 min was given between each trial. Athletes were given verbal encouragement in order to ensure optimal performance. After a maximum of three trials, the 3RM was determined in all individuals. The 1RM was estimated according to Baechle and Earle [32]. The squat movement was performed following the rules of the International Powerlifting Federation [33].

#### Three-Point Explosion

**Fig. 1:**
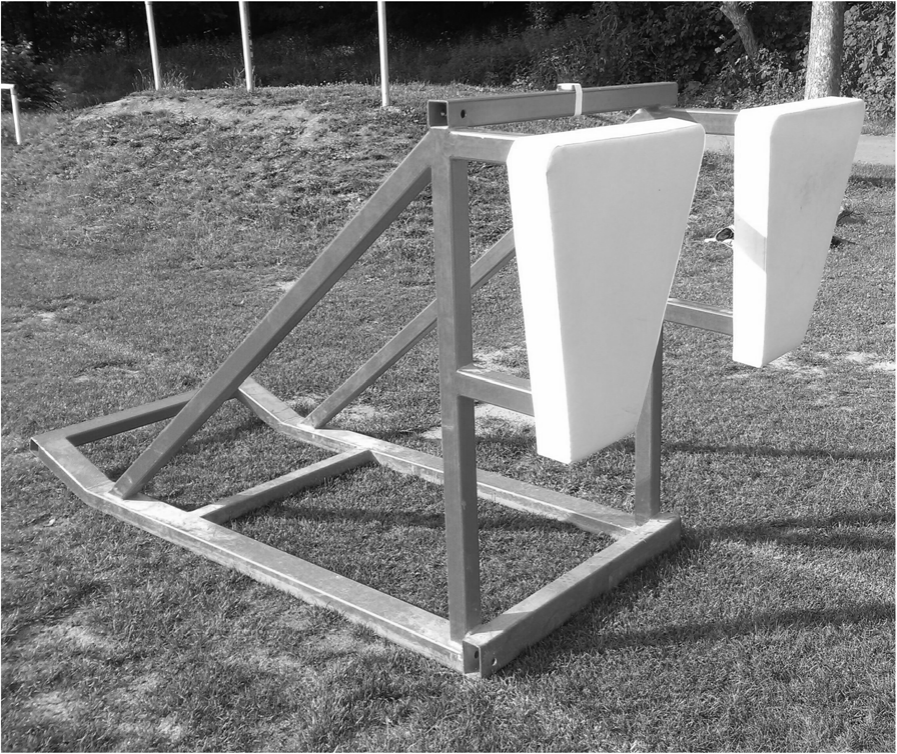
Self-made double blocking sled

The TPE was performed against a self-made double blocking sled (Fig. 1) with a total weight of 150 kg. During the blocking action, the participants were instructed to wear their whole gear including shoulder pad and helmet in order to simulate a realistic situation and avoid injuries. The athletes took a three-point stance with one arm length away from the tackling pad as a standardized position. Their feet were placed approximately shoulder width apart with their toes showing forward to the sled with the dominant foot set back a little. The knuckles of the hand on the side of the dominant foot hit the ground with brunt on it. The other arm lied relaxed in bent position on the thigh. The shoulders were held parallel to the ground, back in a flat position, with the eyes facing toward the target [26]. After the start signal, the athletes exploded by extending their legs and hips and pushing the sled as hard as possible by grabbing the tackling pad with both hands for approximately 2 s. The participants were instructed to attack the left tackling pad only. The acceleration of the sled was measured with a three-dimensional motion tracker (Xsens MTw Awinda, Xsens Technologies B.V., Enschede, Netherlands), which was mounted in the middle of the cross girder. The measurements were performed with a sampling frequency of 100 Hz. Data were extracted for frontal (x-axis), transversal (y-axis) and sagittal (z-axis) acceleration. The three-dimensional sled velocity after 0.5 s (v_0.5s_) and 1 s (v_1s_) was computed (due to numerical integration) using MATLAB (MATrix LABoratory, R2016b (Version 9.1), Mathworks, Inc., Natick, MA, USA).

The athletes were instructed to perform the tests with maximum explosive power and received verbal encouragement by the researcher standing next to the blocking sled. Each participant performed two trials of TPE with a rest interval of 30 s in between. Intra-session reliability of those two trails was calculated using the intraclass correlation coefficient (ICC(2,1)). For both measures the reliability in the TWU condition was excellent (ICC_0.5s_ = 0.930, *p* < 0.001; ICC_1s_ = 0.925, *p* < 0.001). In the PAPE condition, ICC was high for 0.5 s (ICC_0.5s_ = 0.760, *p* = 0.003) but not for 1 s (ICC_1s_ = 0.114, *p* = 0.403).

#### 40-Yard Dash

Infrared timing gates (Brower TCi Gate Timing System, Draper, Utah, USA) were set up at 5, 10 and 36.6 m positions. The participants performed each 40YD from a standardized three-point starting position. The front foot and the opposite hand were placed on the starting line. At the back foot, the timing gates were placed facing the heel of the athlete at approximately 15 cm off the ground. The time set off by releasing the back foot from the sensor. In agreement with Bevan et al. [23] and Turner et al. [14], timing gates were installed at 80 cm off the ground to avoid the breakthrough of the light ray by the lower arm or leg during the sprinting movement. Participants were instructed to perform at maximal effort until passing the last timing gate [14]. Athletes also received verbal encouragement from the researcher at the start and the coach at the finish line. Each athlete was given two trials with a rest period of 2 min. During the rest period, athletes were asked to walk back slowly to the starting point and to wait in a standing position. Intra-session reliabilities of each section (5, 10 and 36.6 m distance) were excellent in the TWU condition (ICC_5m_ = 0.949, *p* < 0.001; ICC_10m_ = 0.970, *p* < 0.001; ICC_36.6m_ = 0.995, *p* < 0.001) as well as in the PAPE condition (ICC_5m_ = 0.926, *p* < 0.001; ICC_10m_ = 0.969, *p* < 0.001; ICC_36.6m_ = 0.996, *p* < 0.001).

### Data analysis

For statistical analyses, the best trial of each test was chosen. Following a test for the normality of distribution (Shapiro-Wilk) that revealed no normal distribution, data was expressed in median (interquartile range). Wilcoxon signed-rank test was used to identify differences between conditions. In the secondary analysis, wide receivers were excluded from calculation because they supposed to have a lower strength level compared to the other positions. Effect size *r* was calculated using z value from Wilcoxon signed-rank test. The cut-off values were defined as around 0.1 for a small effect, around 0.3 for a medium effect and more than 0.5 for a large effect [34]. The level of significance was set at *p* < 0.05. All statistical procedures were performed using SPSS software (Version 20.0; IBM SPSS Statistics for Windows, Chicago, IL, USA).

## Results

### Three-Point Explosion

**Fig. 2:**
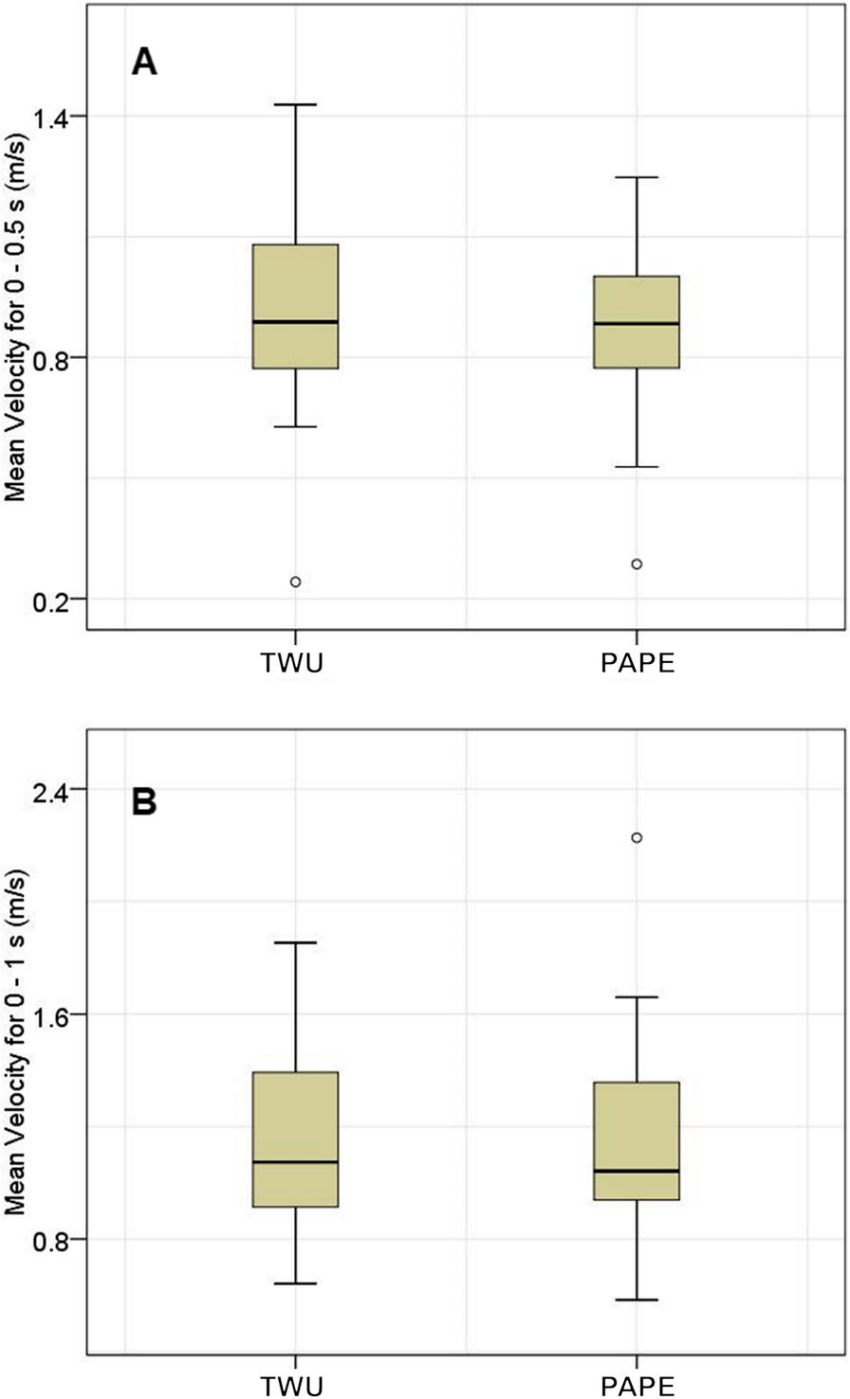
Mean velocity for 0 – 0.5 s (**A**) and 0 – 1 s (**B**) using a traditional warm-up (TWU) and PAPE

There was no significant difference in velocity between the TWU and PAPE conditions after 0.5 s (vTWU_0.5s_ = 0.888 (0.316) m·s^− 1^; vPAP_0.5s_ = 0.884 (0.276) m·s^− 1^; *p* = 0.327) and 1 s (vTWU_1s_ = 1.074 (0.501) m·s^− 1^; vPAP_1s_ = 1.041 (0.462) m·s^− 1^; *p* = 0.586) of the blocking motion (Fig. 2). Furthermore, there were no significant changes related to specific positions.

### 40-Yard Dash

**Fig. 3:**
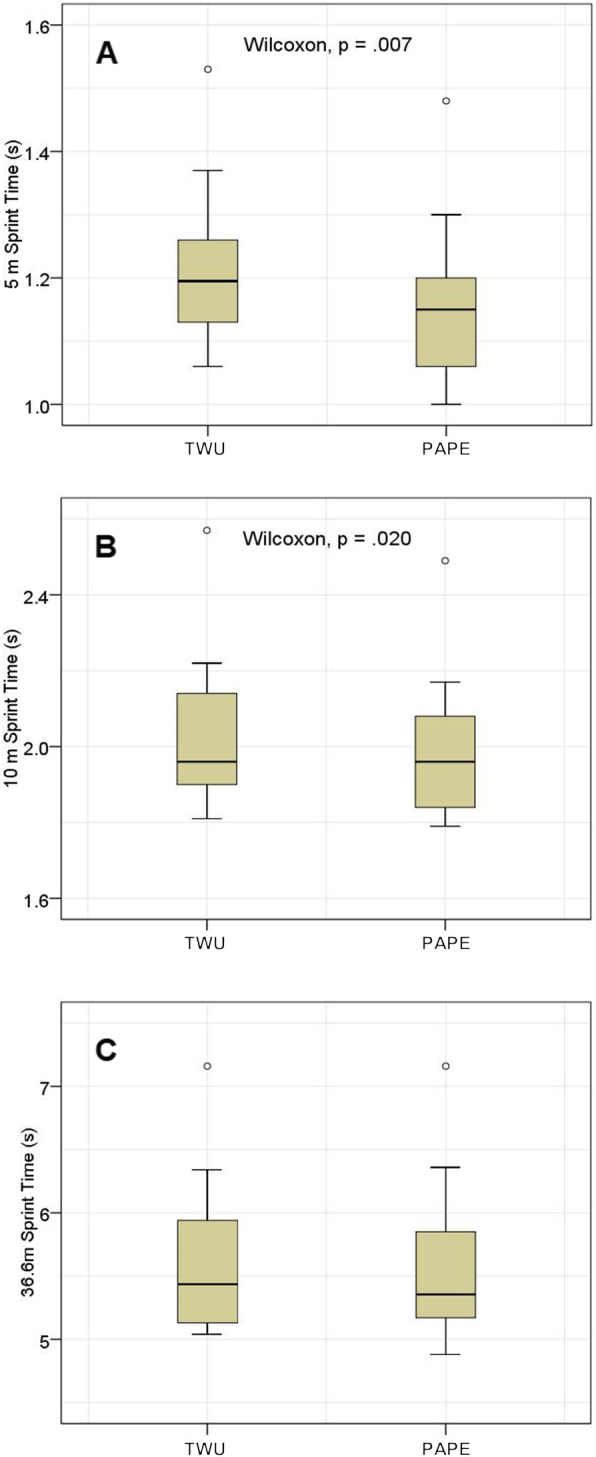
Sprint time for 5 m (**A**), 10 m (**B**) and 36.6 m (**C**) distance in a traditional warm-up (TWU) and PAPE.

The sprinting time was significantly lower with medium effect sizes following the PAPE compared to the TWU protocol for 5 m (tTWU_5m_ = 1.20 (0.16) s; tPAPE_5m_ = 1.15 (0.15) s; *p* = 0.007; *r* = 0.45) and 10 m distance (tTWU_10m_ = 1.96 (0.26) s; tPAPE_10m_ = 1.96 (0.25) s; *p* = 0.020; *r* = 0.39) (Fig. 3). The difference in sprinting time for the whole 36.6 m distance missed the level of significance (tTWU_36.6m_ = 5.44 (0.87) s; tPAPE_36.6m_ = 5.36 (0.72) s; *p* = 0.084; *r* = 0.29). By excluding the wide receivers from the calculation, the sprinting time of the PAPE condition was significantly lower with large effect sizes in all three sections (*p* ≤ 0.005; *r* ≥ 0.57; Table 2).

**Table 2:** Median (interquartile range) in the traditional warm-up (TWU) and PAPE condition, effect size (r) and p-value (p) for 5, 10 and 36.6 m sprinting distance of the 40-yard dash calculated for all participants and without wide receivers.

Sprinting section	All participants (*n* = 18)				Without wide receivers (*n* = 12)			
	**TWU** **Time (s)**	**PAPE** **Time (s)**	***r***	***p***	**TWU** **Time (s)**	**PAPE** **Time (s)**	***r***	***p***
**5 m**	1.20 (0.16)	1.15 (0.15)*	0.45	0.007	1.22 (0.22)	1.15 (0.15)*	0.63	0.002
**10 m**	1.96 (0.26)	1.96 (0.25)*	0.39	0.020	2.03 (0.26)	1.96 (0.31)*	0.63	0.002
**36.6 m**	5.44 (0.87)	5.36 (0.72)	0.29	0.084	5.63 (0.95)	5.44 (0.92)*	0.57	0.005

* *p* < 0.05 (from Wilcoxon signed-rank test)

## Discussion

The aim of the present study was to compare the effects of a heavy load back squat PAPE protocol with a TWU on TPE and 40YD performances in American football players. Partially in agreement with our hypothesis, the PAPE protocol improved the 40YD performance compared to TWU. Sprint times were shorter for 5 and 10 m with medium effect sizes using the PAPE protocol but not for 36.6 m. For the TPE performance, no significant effects were found. According to our results, an almost maximal conditioning contraction, such as a single set of heavy load back squats at 91 % 1RM significantly improved short distance sprint performance. Similar results in sprint performance were shown by Bevan et al. [23] for professional rugby players. Beneficial PAPE effects were also found for the total 40YD distance with large effect sizes when the wide receivers were not considered for data analysis. These findings are in agreement with those of Evetovich et al. [10] who found significantly lower 40-yard sprint time after performing parallel back squat in male NCAA Division II football players. Comparable results were found by Gourgoulis et al. [35], who tested the effect of submaximal half squats on vertical jumping performance. Based on their data they also mentioned greater effects in athletes with a relatively high strength ability [35].

The results of the TPE showed no significant difference between the PAPE and TWU protocol. There are three possible explanations for our findings. First, a meta-analysis of Seitz and Haff [7] showed that less trained athletes need a longer recovery period to dissipate neuromuscular fatigue and to benefit from a potentiation effect. Thus, the lower performance level of our athletes might be responsible for the lack of effects in the chosen time window. In the present study, the TPE was performed twice after in a recovery window of 8 min [13, 22], followed by the 40YD at approximately 10 min recovery time. Therefore, a recovery time of 8 min might have been too short in our athletes. Future studies should therefore focus on the effects after a longer recovery time of ≥ 10 min. This is supported by the results of da Silva Santos et al. [36], who showed a significant greater effect of a conditioning complex consisting of half-squats and plyometric vertical jumps on the number of turning kicks in taekwondo athletes after a resting period of 10 min compared to 5 min or a self-selected period. However, contrary to these findings, the meta-analysis of Wilson et al. [4] reported greater effect sizes when a recovery time of 7 to 10 min instead of > 10 min was used after a conditioning contraction.

Secondly, the lower performance level of included athletes also increases the risk of low or unstable technical skills during the TPE. This questions the applicability of the TPE movement as an adequate test instrument in lower skilled players compared to that with a longer training experience in American football. Another influencing factor could be the playing position. The TPE is predominantly trained and used by linemen [26]. Thus, the technical TPE skills might be lower in players on other playing positions.

A third possible explanation is the complexity of the TPE movement which involves the lower and upper body muscle groups. For example, Evetovich and colleagues [10] examined the effect of PAPE on shot put distance in male and female NCAA Division II shot putters. Comparable to TPE, a shot put combines activities of lower and upper body muscles. The authors [10] found that a PAPE protocol with bench press as an upper body exercise enhanced shot put performance, while a parallel back squat as a the lower body exercise decreased the shot put performance. It might be possible in our study that the upper body (by extending the arms pushing forward to the tackling pad with both hands) was considerably involved in the blocking movement while our PAPE protocol only focused on the muscles of the lower extremities. Therefore, future research is needed to analyze the effect of an upper body PAPE protocol on subsequent TPE performance as well as their underlying physiological mechanisms (e.g. muscle activation, muscle temperature, psychological state) [2, 3].

Study limitations include on the one hand the sample size and the timing of tests. On the other hand, only few experienced American football players can be found in the third division of the German Football league. Most of them have previously participated in other sports and started in their late teenage years or early twenties with regular American football practice and game participation. Due to this, athletes within a team differ regarding technical skills, muscle strength abilities, and therefore the time needed for recovery following a PAPE protocol [18, 20]. Furthermore, the study took place during the regular season with weekly games. This may have caused neuromuscular fatigue, which might have affected our results.

In conclusion, the present study observed increases in performance in 40YD after an almost maximum PAPE protocol (91 % 1RM back squat) compared to an American football specific TWU routine. Especially, within the first meters with a high acceleration (5 and 10 m) the used PAPE protocol appeared to be beneficial. There was no difference in TPE performance between the PAPE and TWU condition. Considering these results, future research should analyze individual responses of players with different skills in order to identify the true benefits of the used PAPE protocol as a warm-up technique before American football games and practice. Further studies should also focus on the optimum timing of recovery following the PAPE protocol.

## Conclusions

The used heavy load back squat PAPE protocol is suitable to increase short distance sprint performance in less experienced American football athletes, while no effect was found on the TPE performance. Muscle strength abilities, game position and recovery time seem to influence the effects and should be carefully considered for the detailed planning of the PAPE procedure.

## Data Availability

The datasets used and/or analyzed during the current study are available from the corresponding author on reasonable request.

## Abbreviations

1RM: One-repetition-maximum
3RM: Three-repetition-maximum
40YD: 40-yard dash
ICC: Intraclass correlation coefficient
PAPE: Postactivation performance enhancement
TPE: Three-point explosion
TWU: Traditional warm-up
